# Prevalence, type of infections and comparative analysis of detection techniques of intestinal parasites in the province of Belgarn, Saudi Arabia

**DOI:** 10.7717/peerj.13889

**Published:** 2022-08-11

**Authors:** Abdulrahman S. Alqarni, Majed H. Wakid, Hattan S. Gattan

**Affiliations:** 1 Health Affairs, Sabt Al-Alaya General Hospital, Directorate of Health Affairs, Ministry of Health, Bisha, Saudi Arabia; 2 Department of Medical Laboratory Technology, Faculty of Applied Medical Sciences, King Abdulaziz University, Jeddah, Saudi Arabia; 3 Special Infectious Agents Unit, King Fahd Medical Research Center, King Abdulaziz University, Jeddah, Saudi Arabia

**Keywords:** Prevalence, Intestinal parasites, Protozoa, Detection techniques, Microscopy, RDTs, Real-time PCR, Food handlers, Belgarn, Saudi Arabia

## Abstract

**Background:**

The study was conducted to observe the prevalence and type of infection caused by intestinal parasites and to compare the techniques that are available for the detection.

**Method:**

A total of 112 stool samples were obtained from study participants, and the laboratory examinations were performed at Special Infectious Agents Unit, King Abdulaziz University, Jeddah. One hundred and twelve participants were included in this study.

**Result:**

The color of positive specimens was mainly brown (86.4%). Stool consistency in infected cases was soft in (64%) samples. There was no statistically significant difference with the physical characteristics of the stool (*P* > 0.05). In total, 59 of the 112 participants were infected with intestinal parasites, representing 52.7%. Different intestinal protozoa parasites were identified in which *Blastocystis hominis* (86.4%) was highest. None of the intestinal helminths were detected. Out of the 59 infected cases, single infections were found in (62.7%) samples. The intestinal protozoan parasites in single infections were *B. hominis* (78.4%), *Giardia lamblia* (8.1%), and (2.7%) from each *Entamoeba histolytica*, *Cryptosporidium parvum*, *Entamoeba coli*, *Endolimax nana*, and *Chilomastix mesnili*. Microscopy, RDTs, and real-time PCR were used for detection and identification of *G. lamblia*, *E. histolytica*, and *C. parvum*.

**Conclusion:**

The study reported a high rate of intestinal parasitic infections, mainly with *B. hominis*. There were no statistical differences in parasite detection between the three techniques in detection of the thirteen cases infected with the pathogenic protozoa.

## Introduction

Intestinal parasitic infections (IPIs) are one of the most common infectious diseases, posing health risks in a variety of countries, particularly in developing countries (Shahnazi et al., 2009). Intestinal parasites may survive, reproduce, and cause pathological symptoms in the human gastrointestinal system. The World Health Organization (WHO) reported that 24% of the population of the world is infected with soil-transmitted helminths (STHs), whereas over 3 billion have no symptoms and more than 800 million children are at risk of exposure (World Health Organization, 2020a). Several serious health problems, such as malnutrition, developmental delay, several forms of anemia, cancer, poor school outcomes and other problems, may result from IPIs (Torgerson et al., 2015).

The prevalence of intestinal parasites in the community has a negative impact on socioeconomic status (Hussain et al., 2019). Developmental disturbances, vitamin A insufficiency, iron deficiency anemia, and weak educational achievement are the consequences of IPIs on children (Crompton & Nesheim, 2002). In addition, IPIs in immunocompromised patients, such as those with HIV, transplant patients and patients with renal dialysis, may lead to serious complications (Omrani et al., 2015). In some cases, parasites may live in the human intestines for years without any symptoms (CDC, 2021).

The most known helminths found in the human intestine include nematodes (roundworms), cestodes (tapeworms) and trematodes (flatworms). The most prevalent causes of IPIs are STHs; *Ascaris lumbricoides*, *Trichuris trichiura*, and hookworms (*Ancylostoma duodenale* and *Necator americanus*). One or more of these helminths infect a large proportion of the global population. *A. lumbricoides* infects approximately 1 billion people every year, *T. trichiura* infects around 700 million, and hookworms infect around 600 million (CDC, 2020; World Health Organization, 2020b). *Enterobius vermicularis*, *Strongyloides stercoralis*, *Taenia spp*., *Schistosoma mansoni* and *Hymenolepis nana* are other important helminths of IPIs.

*Giardia lamblia*, *Entamoeba histolytica*, *Cyclospora cayetanensis*, and *Cryptosporidium parvum* are often the most prevalent pathogenic protozoan parasites in the intestine (Davis, Haque & Petri Jr, 2002). *Blastocystis hominis* is one of the most frequent intestinal protozoans in humans with the greatest prevalence in developing countries. However, there is still much debate about its pathogenicity (Aldahhasi, Toulah & Wakid, 2020; Roberts et al., 2014).

The stool sample is used to identify the occurrence of intestinal parasites diagnostic stages and should be gathered in a clean container, fresh or stored under proper conditions. It is possible to use other samples, such as duodenal aspirates and biopsies, but they are invasive and typically not commonly used for diagnosis of all intestinal parasites. The recommended testing methods may vary according to the suspected disease, travel history, and regional prevalence (CDC, 2018).

Stool specimens should be investigated macroscopically in aspects of color, consistency, existence of blood and mucus, adult worms or proglottids of tapeworms. In addition, stool samples will be examined microscopically by different techniques to identify diagnostic stages of protozoa and helminths.

Fecal antigen-detection tests, commercially available for identification of the three main pathogenic intestinal protozoa (*E. histolytica, G. lamblia* and *Cryptosporidium*).

Molecular methods for different types of intestinal parasites have shown excellent sensitivity and specificity, and the use of such methods in regular laboratory work has been limited. Real-time polymerase chain reaction (PCR) is a highly accurate technique that can detect intestinal parasites even without symptoms (Ten Hove et al., 2009).

This study was conducted for the first time among all food handlers in Belgarn province, Saudi Arabia, to assess the prevalence, type of intestinal parasites, and compare between the performed detection techniques.

## Materials & Methods

### Study area and participants

This study was approved by the research and ethics committee of the Faculty of Applied Medical Sciences at King Abdulaziz University, Jeddah, Saudi Arabia (approval number FAMS-EC2021-08). It was a cross sectional study in which participants were collected from Belgarn province of Saudi Arabia, conducted between April and September 2021. Belgarn is a small province, located in the southwestern part of Saudi Arabia, on mountaintops two thousand feet above sea level. The total area of the province is 1,415 square kilometers, and the population is about 75,000 people.

The total number of food handlers working in the province is 112. All these food handlers were included in the study over the official arrangement and supervision of the Municipality of Belgarn province, without any exclusion criteria.

Simply, a food handler in this study is anyone who works in a food service establishment (restaurants, cafeterias, and coffee shops) and prepares, processes or serves food, or comes into contact with equipment and utensils used to prepare or serve food.

Each participant was provided with a clean labeled plastic container and instructions to collect a stool sample (1- Collect first morning stool directly in the provided labelled clean wide mouth container. 2- Try to fill around a third of the container. 3- The specimen should not be contaminated with water or urine. 4- Close the container tightly. 5- Thoroughly wash hands with soap and water. 6- Place the container in the provided collection bag and keep it in a cool place until obtained from you on the same day).

Each participant signed a consent form as well as filling out a questionnaire to collect data. All collected samples were transported to the microbiology laboratory at Sabt Al-Alaya General Hospital to perform macroscopic examination. For the remaining examinations, samples were transferred to the Special Infectious Agents Unit at King Fahd Medical Research Center, King Abdulaziz University in Jeddah.

In cooperation with the Municipality of Belgarn province, each infected food handler was instructed to visit Sabt Al-Alaya General Hospital for follow up.

### Macroscopic and microscopic examination

The stool samples were investigated macroscopically for color, consistency, the existence of blood and mucus, and for any adult worms or proglottids of tapeworms that could be seen in stool.

Two mg of stool was mixed in 1–2 drops of iodine solution to prepare direct smears. Spread evenly on a microscope slide, covered with a cover glass, and scanned on a light microscope with 10×  and 40×  objective lenses (Garcia et al., 2017).

The sedimentation technique was performed by emulsifying 1–2 g from each stool sample in a 50 ml tube with 10 ml of 10% formal saline solution. The sample was centrifuged at 2,000 rpm for 5 min, then the supernatant was decanted. The sediment was resuspended in 10 ml of 10% formal saline, 3 ml ether was applied, and the mixture was properly shaken before centrifuging for 5 min at 2,000 rpm. The first three layers were removed, and the sediment was investigated under a light microscope after being mixed with two drops of iodine (Garcia et al., 2017).

SDL Quick Trichrome Stain System was used to confirm applicable intestinal protozoan stages. A fecal sample was smeared on a glass slide and allowed to air dry. The slide was dipped four times in 70% ethanol before being immersed in heated trichrome stain for 20 s. Then, the slide was dipped four times in decolorizer and then four times in 90% ethanol. Finally, the smear was dipped four times in xylene, then examined with an oil immersion objective lens (Scientificdevice, 2018).

The modified Kinyoun’s stain was used to detect *Cryptosporidium*. A stool smear was prepared from each sample and allowed to air dry, then fixed with methanol. After that, the smear was stained for 5 min with carbol-fuchsin. Each smear was then washed with tap water, decolorized for 2 min in acid alcohol, washed with tap water, and then counter-stained for 5 min with methylene blue. Finally, the stained smear was washed with tap water, air dried, and examined under the light microscope (Garcia et al., 2017).

### Rapid Diagnostic Tests (RDTs)

The CerTest Biotec rapid card was used to detect *Cryptosporidium* and *Giardia*, while *E. histolytica* was detected by Operon kit. For *Cryptosporidium* and *Giardia*, the stool collection tube cap was removed, and the stick was used to collect enough sample. To ensure good sample dispersion, the tube was shaken. The CerTest Crypto+Giardia combo card test was opened, and four drops were poured into each circular window. The results were read after 10 min and interpreted based on the appearance of the lines (CerTest, 2021).

For the Operon-*E. histolytica* test, 1 ml of extraction buffer was placed in a testing tube, and about 50 mg of stool sample was added with a wooden stick and centrifuged for 5 min at 3,000 rpm after properly shaking the test tube. A total of 125 µl of supernatant was added to the round window of the reaction device, 5 min of incubation at room temperature, and the result was read based on the appearance of the lines (Operon, 2018).

### Molecular Diagnosis (Real-time PCR)

The extracted DNA was used to detect the presence of three pathogenic intestinal parasites: *G. lamblia*, *E. histolytica*, and *C. parvum* using different primer set (Llewellyn et al., 2016; Won et al., 2016), (Table 1). The QIAamp Fast DNA Stool Mini kit was used for DNA extraction from frozen stool specimens stored at −20 °C. Approximately 180–220 mg or 200 µl of watery sample was taken from the stool sample and placed in a 2 ml microcentrifuge tube that was kept on ice. After that, 1 ml of InhibitEX buffer was added to the sample, and the sample was vortexed for 1 min. 1.2 ml of stool lysate was added to a microcentrifuge tube, and the sample was then centrifuged at full speed for 1 min to pellet feces particles. In a new 2 ml microcentrifuge tube that included 25 µl proteinase K, 600 µl supernatant was placed, followed by 600 µl Buffer AL and then vortexed for 15 s. The sample was incubated for 10 min in a water bath at 70 °C, then was shaken briefly to eliminate any droplets from the tube cover. A total of 600 µl lysate was properly applied to the QIAamp spin column and was centrifuged at full speed for 1 min. The QIAamp spin column was transferred into a new 2 ml collecting tube. Following that, 500 µl of AW1 buffer was placed to the QIAamp spin column and was centrifuged for 1 min at full speed. After centrifugation, the QIAamp spin column was transferred into a new collecting tube. Following that, 500 µl AW2 buffer was gently placed to the QIAamp spin column and was centrifuged for 3 min at full speed. The QIAamp spin column was then transferred to a new, labeled 1.5 ml microcentrifuge tube, and 200 µl buffer ATE was added immediately onto the QIAamp membrane. Lastly, after 1 min of room temperature incubation, centrifugation at full speed for 1 min was used to elute the DNA. The extracted DNA samples were kept at −20 °C for later use (QIAamp, 2020).

**Table 1:** Real-time PCR primers and probes. This table shows the used primers and probes with real-time PCR.

Gene	Primers and sequence (5′to 3′)	Reference
*G. lamblia* Beta- giardin	F-(5′-GAG GTC AAG AAG TCC GCC G-3′) R-(5′-CAA GGG ACT TGC GGA AGT TT-3′) P-(5′-ACG ATC AAG GAG GAG ATC GA-3′)	Won et al. (2016)
*E. histolytica* SSU rRNA	F-(5′-AAC AGT AAT AGT TTC TTT GGT TAG TAAAA-3′) R-(5′-CTT AGA ATG TCA TTT CTC AAT TCA T-3′) P- (5′-ATT AGT ACA AAA TGG CCA ATT CAT TCA-3′)	Llewellyn et al. (2016)
*C. parvum* Cowp 1	F-(5′-TGT GTT CAA TAT CTC CCT GCA AA-3′) R-(5′-GCA TGT CGA TTC AAT TTG TCA-3′) P-(5′-CCT CCT GGA TTC AAT TTG TCA-3′)	Won et al. (2016)

For real-time PCR amplification, primer and probe were diluted by adding 10 µl of stock to a new labeled 1.5 microcentrifuge tube, followed by 90 µl of RNase/DNase free water to make a final volume of 100 µl (dilution factor = 1:10). The master mix was made in a 1.5 ml microcentrifuge tube based on the QuantiTect^®^ Probe PCR kit guidelines, then was vortexed thoroughly, and finally was centrifuged at full speed. The reaction mix was then transferred in the required amount (15.5 µl) to a standard PCR plate, and 4.5 µl of pure DNA was placed to each well (Table 2). As a negative control, a PCR reaction with no DNA template (just RNase/DNase free water) was performed for every run. The plate was centrifuged for 1 min to mix the components. To begin the cycling program, the plate was placed in an Applied Biosystems 7500 Fast cycler, and the condition was set in accordance with the QuantiTect^®^ Probe PCR kit’s guidelines (QuantiTect, 2011). The Applied Biosystems 7500 Fast System was used to analyze the data, which computed the Ct value (threshold cycle number) for positive gene amplification in real-time-cycler number.

**Table 2:** Real-time PCR components for gene amplification. This table shows Real-time PCR components for gene amplification, including the amount of each component.

**Component**	**Amounts in **µ** l**
2× QuantiTect Probe PCR master mix	6.5
RNase-free water	4.5
Forward	1.5
Reverse	1.5
Prob	1.5
DNA	4.5
Total	20

### Statistical analysis

The data collected was analyzed using the Statistical Package for the Social Sciences (the IBM SPSS^®^ version 25 statistical software). The descriptive analysis was used to evaluate demographic data and categorical variables. All reported *P* values were two-tailed and *P* < 0.05 is considered significant.

## Results

**Figure 1:**
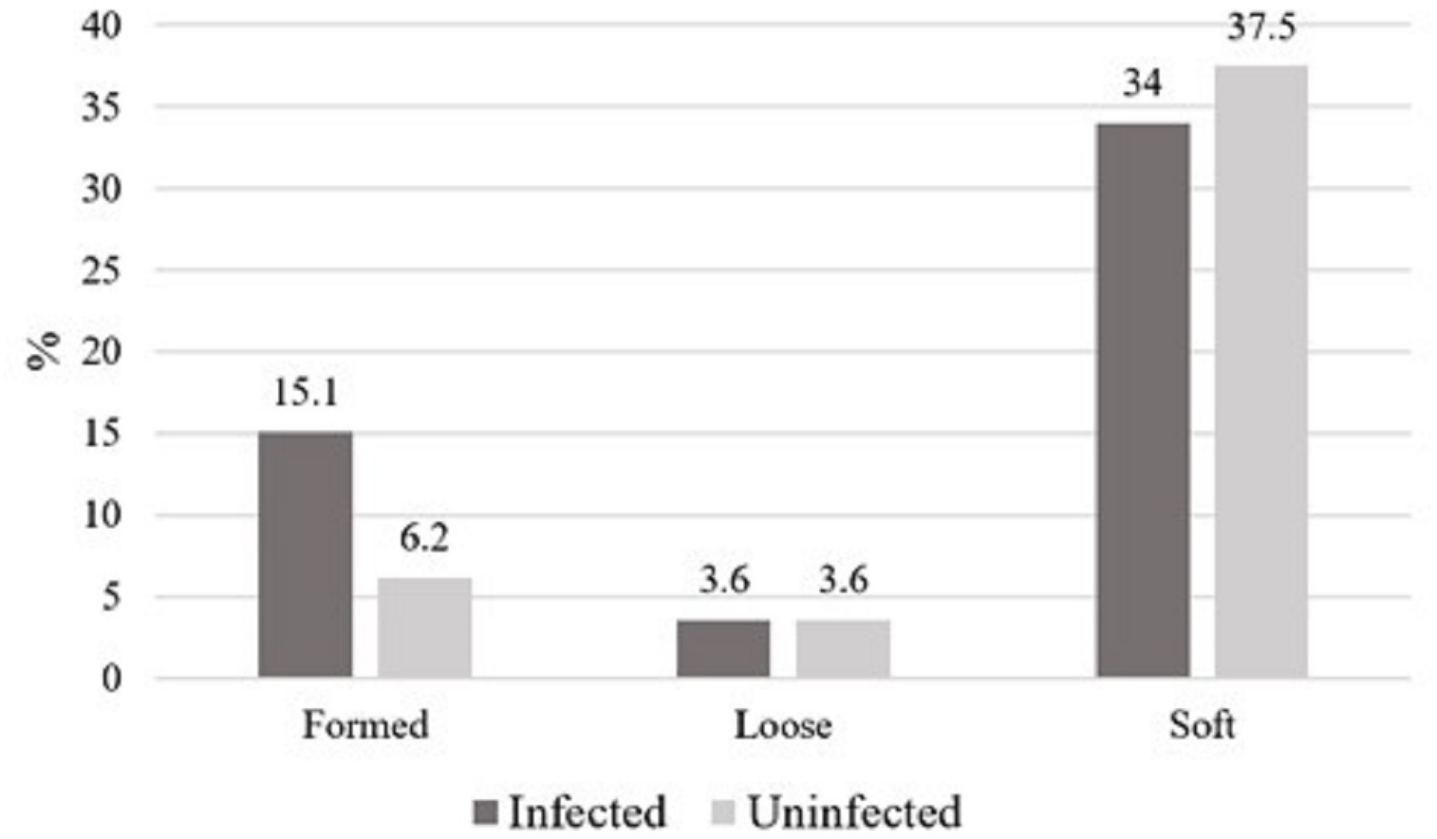
The consistency of stool specimens in all infected cases with pathogenic and nonpathogenic parasites and uninfected cases, represented as percentage. This figure shows the consistency of stool specimens in all infected cases with pathogenic and nonpathogenic parasites and uninfected cases. the data are represented as percentage.

One hundred and twelve participants ranging in age from 20 to 65 years old (33.5 ± 9.2) were included in this study. Information on socio-demographic status, nationality, level of education, symptoms and hygienic habits is being presented as part of a separate study and is not included here. The color of positive specimens ranged from brown (51, 86.4%) to dark brown (8, 13.6%). Stool consistency in infected cases including pathogenic and nonpathogenic parasites was soft in 38 samples, formed in 17, and loose in four, whereas consistency in uninfected cases was soft in 42 samples, formed in seven, and loose in four (Fig. 1). There were no adult worms or proglottids of tapeworms in any of the investigated samples. There was no statistically significant difference with the physical characteristics of the stool.

In total, 59 of the 112 participants were infected with intestinal parasites, representing 52.7%. Using microscopic examinations, different intestinal protozoa parasites were identified such as: *B. hominis* 51 (86.4%), *Entamoeba coli* 13 (22%), *E. nana* 11 (18.6%), *G. lamblia* 6 (10.2%), *E. histolytica* 6 (10.2%), *C. mesnili* 4 (6.8%), *Iodamoeba buetschlii* 1 (1.7%), and *C. parvum* 1 (1.7%). Using microscopy, no eggs or other diagnostic stages of intestinal helminths were detected.

Out of the 59 infected cases, single infections were found in 37 (62.7%) samples, double infections in 13 (22%) samples, triple infections in 7 (11.9%) samples, quadruple infections in one (1.7%) sample, and quintuple infections in one (1.7%) sample. The intestinal protozoan parasites in single infections were *B. hominis* (29, 78.4%), followed by *G. lamblia* (three, 8.1%), and one (2.7%) from each *E. histolytica*, *C. parvum*, *E. coli*, *E. nana*, and *C. mesnili*. Table 3 lists the parasites found in each infection type (single, double, triple, quadruple, and quintuple) by microscopic examination.

**Table 3:** The prevalence of the detected parasites in each infection type, using microscopy. The prevalence of all detected parasites (pathogenic and nopathopgenic) in each infection type, using microscopic examination techniques.

**Type of infection**	**No.**
Single infection (*n* = 37, 62.7%^*^)	
*B. hominis*	29
*G. lamblia*	3
*E. histolytica*	1
*C. parvum*	1
*E. coli*	1
*E. nana*	1
*C. mesnili*	1
Double infection (*n* = 13, 22%^*^)	
*B. hominis + E. nana*	5
*B. hominis + E. coli*	4
*B. hominis + G. lamblia*	2
*B. hominis + C. mesnili*	1
*B. hominis + E. histolytica*	1
Triple infection (*n* = 7, 11.9%^*^)	
*B. hominis + E. coli + E. histolytica*	2
*B. hominis + E. coli + E. nana*	2
*B. hominis + E. nana + G. lamblia*	1
*B. hominis + E. coli + I. buetschlii*	1
*B. hominis + E. coli + C. mesnili*	1
Quadruple infection (*n* = 1, 1.7%^*^)	
*B. hominis + E. coli + E. nana + E. histolytica*	1
Quintuple infection (*n* = 1, 1.7%^*^)	
*B. hominis + E. coli + E. nana + C. mesnili + E. histolytica*	1

**Notes.**

* % Calculated to the total number of the infected cases (59).

Pathogenic intestinal protozoa *G. lamblia*, *E. histolytica*, and *C. parvum* were identified using microscopy, RDTs, and real-time PCR. There were no differences in parasite detection between the three techniques for *G. lamblia* (6, 10.2%) and *C. parvum* (1, 1.7%). For *E. histolytica* (6, 10.2%), RDTs detected parasites in only three samples, whereas real-time PCR identified *E. histolytica* in all six samples. There was no statistically significant difference between the techniques used (*P* = 0.53) for detection of the pathogenic protozoa (Table 4). The infected samples with *G.lamblia* (*n* = 6), *E. histolytica* (*n* = 6), and *Cryptosporidium* (*n* = 1) are not the same samples.

**Table 4:** The frequency of detected pathogenic intestinal protozoa (in different samples for each parasite), using different techniques. This table shows the frequency of the detected pathogenic intestinal protozoa using different techniques. The infected samples with *G. lamblia* (*n* = 6), *E. histolytica* (*n* = 6), and *Cryptosporidium* (*n* = 1) are not the same samples. There was no statistical difference between the three techniques in detection of all parasites together in the thirteen samples. The results of the 3 tests were in perfect agreement for *G. lamblia* and *C. parvum*. For *E. histolytica*, the results of microscopy and real-time PCR were in perfect agreement, whereas the RDT was positive for three of the samples found to be positive by the other methods but negative in the other three samples found to be positive by the other methods.

**Pathogenic intestinal parasites**	**Microscopy**	**RDTs**	**Real-time PCR**	*P*-value
*G. lamblia*	6	6	6	–
*E. histolytica*	6	3	6	0.53
*C. parvum*	1	1	1	–

## Discussion

Parasitic diseases typically result in high disease burdens and are often spread by contaminated food to humans. Foodborne pathogens are important globally and contribute to higher morbidity and mortality in Belgarn province. Previous studies on food handlers in Saudi Arabia revealed varying rates of IPIs prevalence. These were 50.15% in Jeddah, 23% in Makkah, 18.8% in Al-Madina, and 22.8% in Bahrah (Wakid, 2006; Zaglool et al., 2011; Taha, Soliman & Banjar, 2013; Wakid, 2020).

The most frequent parasites identified in our study were *B. hominis* (86.4%), *E. coli* (22%), *E. nana* (18.6%), *G. lamblia* (10.2%), and *E. histolytica* (10.2%). According to the study in Bahrah, the most prevalent isolated parasites were *T. trichiura*, *B. hominis*, and *E. nana* (Wakid, 2020). In Al-Madina study, the most prevalent parasites were *G. lamblia*, *E. histolytica/E. coli*, and *T. trichiura* (Taha, Soliman & Banjar, 2013). In the Makkah study, the most prevalent organisms were *G. lamblia*, *E. histolytica/dispar*, and *C. parvum*, while in Jeddah were *B. hominis*, hookworms, and *T. trichiura* (Wakid, 2006; Zaglool et al., 2011).

Direct methods, concentration methods, permanent stains, RDTs, and real-time PCR were all used in this study. Iodine is used in direct smear examination to detect the details of diagnostic features of different parasites. As a result, direct smear examination is regarded as the most suitable and simple method of detecting intestinal parasites. On the other hand, direct smear examination may produce false negative results in cases of low infection levels, requiring the use of more accurate methods. Several concentration methods are commonly used to improve the possibility of detecting parasitic diagnostic stages. One of the most useful concentration techniques for detecting cysts, ova, and larvae in large quantities of stool is the Ritchie sedimentation method. The diagnostic stages are killed and preserved using formal saline, while diethyl ether separates the undesirable debris at the top of the tube and concentrates all the diagnostic stages at the bottom. On the other hand, trophozoites are undetectable due to their adherence to debris, and the chemicals required in this method are carcinogenic and flammable.

Permanent trichrome and modified Kinyoun’s stains were used in this study. Trichrome is regarded as the most suitable stain for detecting cyst and trophozoite morphological characteristics. Because wet mount examinations could miss small protozoa, trophozoites quickly perish, and immediate processing of samples is difficult, staining methods are the best choice. The modified Kinyoun’s stain can be used to identify coccidian oocysts such as *Cryptosporidium*, *Cystoisospora*, and *Cyclospora* from fresh or preserved stool samples that are difficult to identify using routine stains (Wakid, 2006; Zaglool et al., 2011; Taha, Soliman & Banjar, 2013; Wakid, 2020). RDTs use monoclonal antibodies inserted into a device to identify one or more antigens. Two commercial kits were used in this study: the CerTest Crypto+Giardia combo card test and the Operon-*Entamoeba* test. These chromatographic immunoassay kits for detecting three primary protozoan-induced diarrheal disorders. Antigen detection techniques are simple to use and do not require the assistance of a qualified microscopist. Most kits require fresh or preserved stool specimens for antigen detection methods (Wakid, 2006; Zaglool et al., 2011; Taha, Soliman & Banjar, 2013; Wakid, 2020).

As a molecular technique, real-time PCR was used in this study to confirm isolated parasites. For detecting parasites in stool specimens, real-time PCR was found to be superior to microscopic investigation. These methods have the advantage of being able to identify low parasite levels. On the other hand, the high risk of contamination and the high cost of reagents are significant disadvantages. In the present study, 59 participants were infected with either a single parasite or multiple parasites. This is consistent with prior studies in Saudi Arabia, which found that infection rate with a single parasite was higher than multiple parasites (Wakid, 2006; Zaglool et al., 2011; Taha, Soliman & Banjar, 2013; Wakid, 2020). All detected intestinal parasites in our study were protozoa. According to previous studies in Saudi Arabia, protozoa were the most frequently detected parasites (Wakid, 2006; Zaglool et al., 2011; Taha, Soliman & Banjar, 2013; Wakid, 2020). The reason for that is the simple route of protozoa transmission. Additionally, the high prevalence of protozoa can be explained by their high proliferation, ability to form cysts, and resistance to environmental factors (Heydari-Hengami et al., 2018). Pathogenic and opportunistic protozoan parasites were identified in approximately 22.1% of the isolated organisms.

These protozoa were *G. lamblia* (10.2%), *E. histolytica* (10.2%), and *C. parvum* (1.7%). There were no abdominal symptoms in 84.6% of those infected with these protozoa. Asymptomatic infected food handlers pose a significant risk of spreading the infection to their coworkers and customers. In comparison to other studies, the current study found that parasitic infection was more prevalent in Belgarn province. The study also revealed that non-pathogenic parasites accounted for 49.1%, including *E. coli* (22%), *E. nana* (18.6%), *C. mesnili* (6.8%), and *I. buetschlii* (1.7%). Infection with non-pathogenic parasites indicates a lack of hygiene, even though these parasites typically cause no symptoms. The fecal-oral route of transmission of nonpathogenic intestinal protozoa is similar to the pathogenic organism’s transmission, so should not be neglected (Poulsen & Stensvold, 2016; Pagheh et al., 2018).

According to our study, the most common isolated parasite was *B. hominis* (86.4%) from all infected food handlers. Using direct iodine smear, Ritchie concentration technique, and permanent trichrome stain, 51 samples were positive for *B. hominis*. This finding was consistent with previous studies, which found *B. hominis* to be the most isolated parasite, such as a study conducted in Jeddah (23.29%), and Iran (24.3%) (Wakid, 2006; Heydari-Hengami et al., 2018). Since many clinical laboratories do not report or identify this parasite, its prevalence could be higher than revealed in several studies. The pathogenicity of this parasite is still highly debated (Yason et al., 2019; Aldahhasi, Toulah & Wakid, 2020). In the future, we intend to use molecular techniques to obtain more information about the genetic diversity of the isolated *Blastocystis* spp. *B. hominis* has the possibility to cause disease, therefore, it is essential to pay greater attention where further research is needed.

According to isolated parasites, one participant from Bangladesh was infected with *C. parvum*. The result was obtained using RDT, modified Kinyoun’s technique, and real- time PCR. *G. lamblia* was detected in six (10.2%) of participants with or without other parasites. The results were obtained through direct iodine smear, formalin- ether method, RDT, and real-time PCR. All these techniques showed no variations in parasite detection. All *G. lamblia*-infected food handlers claimed that they have no abdominal symptoms. In comparison to previous studies, *G. lamblia* was the most frequently detected intestinal parasite in Makkah study (9%), while Jeddah study revealed 4.6% (Wakid, 2006; Zaglool et al., 2011). A study in Iran found that *Giardia* parasites in food handlers was responsible for the majority of infections (63.37%) (Balarak et al., 2016).

*E. histolytica* was detected in six (10.2%) of participants with or without other parasites. Microscopic examinations, RDTs, and real-time PCR were used to obtain the results. Microscopic examination of amebiasis is somewhat complicated due to the difficulty in distinguishing *E. histolytica* and *E. dispar* due to their morphological similarity. The detection of parasites differed between RDTs and real-time PCR. This is due to RDTs sensitivity and specificity (Vanden Bossche et al., 2015), as only three samples were positive by RDTs and the remaining three were negative, whereas real-time PCR confirmed *E. histolytica* in all six samples. Compared to other studies, the prevalence of *E. histolytica* in Makkah and Jeddah were (4.5%) and (2.97%), respectively (Wakid, 2006; Zaglool et al., 2011).

In an Ethiopian study, *E. histolytica/dispar* was found to be the second most prevalent intestinal parasite (24.4%) (Yeshanew, Tadege & Abamecha, 2021), due to sociodemographic differences as well as the diagnostic techniques used.

Intestinal helminths were not detected in our study because the incidence of intestinal parasites differs based on the geographical and climatic conditions of the region.

## Conclusions

The transmission of intestinal parasites among food handlers could pose a threat to the community. In this study, food handlers had a high prevalence of intestinal parasites. Among infected food handlers, 62.7% were infected with a single parasite, while 37.3% were infected with multiple parasites. There were no helminths detected, only intestinal protozoa, which is still a significant issue among food handlers. Therefore, continuous epidemiological surveillance through regular surveys is required to address the incidence of IPIs. During pre-employment procedures, almost only one specimen is provided for stool analysis. Based on our study findings, several initiatives can be implemented to improve work performance and parasite identification sensitivity. We recommend technologist training, conducting concentration methods, and introducing other techniques such as antigen detection and molecular assays.

##  Supplemental Information

10.7717/peerj.13889/supp-1 Supplemental Information 1 Raw data of the main results for each foodhandler

## Additional Information and Declarations

### Data Availability

The following information was supplied regarding data availability: Raw data are available as a Supplementary File.
