# Supplementary material for: Prevalence, type of infections and comparative analysis of detection techniques of intestinal parasites in the province of Belgarn, Saudi Arabia
Source: PeerJ. 2022 Aug 11;10:e13889. doi: 10.7717/peerj.13889 (PMC9375965; doi:10.7717/peerj.13889)
Supplement: Supplemental Information 1 [file peerj-10-13889-s001.docx]

**Raw Data (1=positive, 2= Negative)**

|  |  |  |  |  | ***Detected parasites by Microscopic Examinations*** | | | | | | | |  |
| --- | --- | --- | --- | --- | --- | --- | --- | --- | --- | --- | --- | --- | --- |
| **Sp.**  **#** | **Color** | **Consistency** | **RDT** | **Real-time PCR** | ***B.***  ***hominis*** | ***E.***  ***coli*** | ***E.***  ***nana*** | ***C.***  ***mesnili*** | ***I.***  ***buetschlii*** | ***G.***  ***lamblia*** | ***E.***  ***histolytica*** | ***Crypto*** | **Types of infection** |
| 1 | Brown | Soft |  |  | 2 | 2 | 2 | 2 | 2 | 2 | 2 | 2 | No infection |
| 2 | Brown | Soft |  |  | 2 | 2 | 2 | 2 | 2 | 2 | 2 | 2 | No infection |
| 3 | Brown | Soft |  |  | 2 | 2 | 2 | 2 | 2 | 2 | 2 | 2 | No infection |
| 4 | Brown | Soft |  |  | 2 | 1 | 2 | 2 | 2 | 2 | 2 | 2 | Single |
| 5 | Brown | Soft |  |  | 1 | 1 | 2 | 2 | 1 | 2 | 2 | 2 | Triple |
| 6 | Brown | Soft |  |  | 2 | 2 | 2 | 2 | 2 | 2 | 2 | 2 | No infection |
| 7 | Brown | Soft | *G. lamblia* | *G. lamblia* | 2 | 2 | 2 | 2 | 2 | 1 | 2 | 2 | Single |
| 8 | Brown | Soft |  |  | 2 | 2 | 2 | 2 | 2 | 2 | 2 | 2 | No infection |
| 9 | Brown | Soft | *E. histolytica* | *E. histolytica* | 2 | 2 | 2 | 2 | 2 | 2 | 1 | 2 | Single |
| 10 | Brown | Soft |  |  | 1 | 2 | 2 | 1 | 2 | 2 | 2 | 2 | Double |
| 11 | Brown | Soft |  |  | 2 | 2 | 2 | 2 | 2 | 2 | 2 | 2 | No infection |
| 12 | Brown | Soft | *E. histolytica* | *E. histolytica* | 1 | 1 | 1 | 2 | 2 | 2 | 1 | 2 | Quadruple |
| 13 | Brown | Formed | *G. lamblia* | *G. lamblia* | 1 | 2 | 2 | 2 | 2 | 1 | 2 | 2 | Double |
| 14 | Brown | Loose |  |  | 1 | 2 | 2 | 2 | 2 | 2 | 2 | 2 | Single |
| 15 | D. brown | Formed |  |  | 2 | 2 | 2 | 2 | 2 | 2 | 2 | 2 | No infection |
| 16 | Brown | Soft |  |  | 1 | 2 | 2 | 2 | 2 | 2 | 2 | 2 | Single |
| 17 | D. brown | Soft |  |  | 2 | 2 | 2 | 1 | 2 | 2 | 2 | 2 | Single |
| 18 | Brown | Formed |  |  | 1 | 2 | 1 | 2 | 2 | 2 | 2 | 2 | Double |
| 19 | D. brown | Formed |  |  | 2 | 2 | 2 | 2 | 2 | 2 | 2 | 2 | No infection |
| 20 | Brown | Soft |  |  | 2 | 2 | 1 | 2 | 2 | 2 | 2 | 2 | Single |
| 21 | Brown | Loose |  |  | 1 | 1 | 2 | 2 | 2 | 2 | 2 | 2 | Double |
| 22 | Brown | Formed |  |  | 1 | 2 | 2 | 2 | 2 | 2 | 2 | 2 | Single |
| 23 | Brown | Soft |  |  | 1 | 2 | 2 | 2 | 2 | 2 | 2 | 2 | Single |
| 24 | D. brown | Soft |  |  | 2 | 2 | 2 | 2 | 2 | 2 | 2 | 2 | No infection |
| 25 | Brown | Soft |  |  | 2 | 2 | 2 | 2 | 2 | 2 | 2 | 2 | No infection |
| 26 | Brown | Formed |  |  | 1 | 2 | 2 | 2 | 2 | 2 | 2 | 2 | Single |
| 27 | Brown | Formed |  |  | 1 | 1 | 2 | 2 | 2 | 2 | 2 | 2 | Double |
| 28 | Brown | Formed |  |  | 1 | 2 | 2 | 2 | 2 | 2 | 2 | 2 | Single |
| 29 | Brown | Soft | *E. histolytica* | *E. histolytica* | 1 | 2 | 2 | 2 | 2 | 2 | 1 | 2 | Double |
| 30 | Brown | Soft |  |  | 1 | 2 | 2 | 2 | 2 | 2 | 2 | 2 | Single |
| 31 | Brown | Soft |  |  | 1 | 2 | 2 | 2 | 2 | 2 | 2 | 2 | Single |
| 32 | Brown | Soft |  |  | 2 | 2 | 2 | 2 | 2 | 2 | 2 | 2 | No infection |
| 33 | Brown | Formed |  |  | 1 | 1 | 2 | 2 | 2 | 2 | 2 | 2 | Double |
| 34 | Brown | Soft |  |  | 2 | 2 | 2 | 2 | 2 | 2 | 2 | 2 | No infection |
| 35 | Brown | Soft |  |  | 2 | 2 | 2 | 2 | 2 | 2 | 2 | 2 | No infection |
| 36 | Brown | Formed |  |  | 2 | 2 | 2 | 2 | 2 | 2 | 2 | 2 | No infection |
| 37 | Brown | Soft | *G. lamblia* | *G. lamblia* | 1 | 2 | 1 | 2 | 2 | 1 | 2 | 2 | Triple |
| 38 | Brown | Soft |  |  | 2 | 2 | 2 | 2 | 2 | 2 | 2 | 2 | No infection |
| 39 | Brown | Soft |  |  | 2 | 2 | 2 | 2 | 2 | 2 | 2 | 2 | No infection |
| 40 | Brown | Soft |  |  | 2 | 2 | 2 | 2 | 2 | 2 | 2 | 2 | No infection |
| 41 | Brown | Soft |  |  | 1 | 2 | 2 | 2 | 2 | 2 | 2 | 2 | Single |
| 42 | Brown | Formed |  |  | 1 | 2 | 1 | 2 | 2 | 2 | 2 | 2 | Double |
| 43 | Brown | Loose |  |  | 2 | 2 | 2 | 2 | 2 | 2 | 2 | 2 | No infection |
| 44 | Brown | Soft |  |  | 1 | 1 | 2 | 1 | 2 | 2 | 2 | 2 | Triple |
| 45 | Brown | Formed |  |  | 2 | 2 | 2 | 2 | 2 | 2 | 2 | 2 | No infection |
| 46 | Brown | Soft |  | *E. histolytica* | 1 | 1 | 2 | 2 | 2 | 2 | 1 | 2 | Triple |
| 47 | Brown | Soft |  |  | 1 | 2 | 2 | 2 | 2 | 2 | 2 | 2 | Single |
| 48 | Brown | Soft |  |  | 2 | 2 | 2 | 2 | 2 | 2 | 2 | 2 | No infection |
| 49 | Brown | Soft |  |  | 2 | 2 | 2 | 2 | 2 | 2 | 2 | 2 | No infection |
| 50 | Brown | Formed |  |  | 1 | 2 | 2 | 2 | 2 | 2 | 2 | 2 | Single |
| 51 | Brown | Loose |  |  | 2 | 2 | 2 | 2 | 2 | 2 | 2 | 2 | No infection |
| 52 | Brown | Soft |  |  | 2 | 2 | 2 | 2 | 2 | 2 | 2 | 2 | No infection |
| 53 | Brown | Soft |  |  | 2 | 2 | 2 | 2 | 2 | 2 | 2 | 2 | No infection |
| 54 | Brown | Soft |  |  | 2 | 2 | 2 | 2 | 2 | 2 | 2 | 2 | No infection |
| 55 | Brown | Formed |  |  | 2 | 2 | 2 | 2 | 2 | 2 | 2 | 2 | No infection |
| 56 | Brown | Soft |  |  | 1 | 2 | 2 | 2 | 2 | 2 | 2 | 2 | Single |
| 57 | Brown | Formed |  |  | 2 | 2 | 2 | 2 | 2 | 2 | 2 | 2 | No infection |
| 58 | Brown | Soft |  |  | 1 | 2 | 2 | 2 | 2 | 2 | 2 | 2 | Single |
| 59 | Brown | Soft |  |  | 2 | 2 | 2 | 2 | 2 | 2 | 2 | 2 | No infection |
| 60 | Brown | Soft |  |  | 2 | 2 | 2 | 2 | 2 | 2 | 2 | 2 | No infection |
| 61 | Brown | Formed |  |  | 1 | 2 | 2 | 2 | 2 | 2 | 2 | 2 | Single |
| 62 | Brown | Soft |  |  | 1 | 2 | 2 | 2 | 2 | 2 | 2 | 2 | Single |
| 63 | Brown | Soft |  |  | 2 | 2 | 2 | 2 | 2 | 2 | 2 | 2 | No infection |
| 64 | Brown | Soft |  |  | 1 | 2 | 2 | 2 | 2 | 2 | 2 | 2 | Single |
| 65 | D. brown | Formed |  |  | 1 | 2 | 2 | 2 | 2 | 2 | 2 | 2 | Single |
| 66 | Brown | Soft |  |  | 1 | 2 | 2 | 2 | 2 | 2 | 2 | 2 | Single |
| 67 | Brown | Soft |  | *E. histolytica* | 1 | 1 | 1 | 1 | 2 | 2 | 1 | 2 | Quintuple |
| 68 | Brown | Soft |  |  | 1 | 2 | 2 | 2 | 2 | 2 | 2 | 2 | Single |
| 69 | Brown | Formed |  |  | 1 | 2 | 2 | 2 | 2 | 2 | 2 | 2 | Single |
| 70 | Brown | Soft |  |  | 2 | 2 | 2 | 2 | 2 | 2 | 2 | 2 | No infection |
| 71 | Brown | Soft |  |  | 1 | 2 | 1 | 2 | 2 | 2 | 2 | 2 | Double |
| 72 | Brown | Soft |  |  | 1 | 1 | 2 | 2 | 2 | 2 | 2 | 2 | Double |
| 73 | Brown | Soft |  |  | 2 | 2 | 2 | 2 | 2 | 2 | 2 | 2 | No infection |
| 74 | Brown | Soft |  |  | 2 | 2 | 2 | 2 | 2 | 2 | 2 | 2 | No infection |
| 75 | D. brown | Formed |  |  | 1 | 2 | 2 | 2 | 2 | 2 | 2 | 2 | Single |
| 76 | D. brown | Formed |  |  | 1 | 2 | 2 | 2 | 2 | 2 | 2 | 2 | Single |
| 77 | Brown | Soft | *G. lamblia* |  | 1 | 2 | 2 | 2 | 2 | 1 | 2 | 2 | Double |
| 78 | Brown | Soft |  |  | 2 | 2 | 2 | 2 | 2 | 2 | 2 | 2 | No infection |
| 79 | Brown | Soft |  |  | 2 | 2 | 2 | 2 | 2 | 2 | 2 | 2 | No infection |
| 80 | D. brown | Soft |  |  | 1 | 2 | 1 | 2 | 2 | 2 | 2 | 2 | Double |
| 81 | D. brown | Soft |  |  | 1 | 2 | 2 | 2 | 2 | 2 | 2 | 2 | Single |
| 82 | D. brown | Formed |  |  | 1 | 2 | 2 | 2 | 2 | 2 | 2 | 2 | Single |
| 83 | Brown | Formed |  |  | 1 | 2 | 2 | 2 | 2 | 2 | 2 | 2 | Single |
| 84 | Brown | Soft |  |  | 2 | 2 | 2 | 2 | 2 | 2 | 2 | 2 | No infection |
| 85 | D. brown | Soft |  | *E. histolytica* | 1 | 1 | 2 | 2 | 2 | 2 | 1 | 2 | Triple |
| 86 | Brown | Soft |  |  | 2 | 2 | 2 | 2 | 2 | 2 | 2 | 2 | No infection |
| 87 | Brown | Soft |  |  | 2 | 2 | 2 | 2 | 2 | 2 | 2 | 2 | No infection |
| 88 | Brown | Soft |  |  | 1 | 1 | 1 | 2 | 2 | 2 | 2 | 2 | Triple |
| 89 | Brown | Soft | *G. lamblia* |  | 2 | 2 | 2 | 2 | 2 | 1 | 2 | 2 | Single |
| 90 | Brown | Soft |  |  | 2 | 2 | 2 | 2 | 2 | 2 | 2 | 2 | No infection |
| 91 | Brown | Soft |  |  | 1 | 2 | 2 | 2 | 2 | 2 | 2 | 2 | Single |
| 92 | Brown | Soft |  |  | 1 | 2 | 2 | 2 | 2 | 2 | 2 | 2 | Single |
| 93 | Brown | Soft |  |  | 1 | 2 | 2 | 2 | 2 | 2 | 2 | 2 | Single |
| 94 | Brown | Soft |  |  | 2 | 2 | 2 | 2 | 2 | 2 | 2 | 2 | No infection |
| 95 | Brown | Loose |  |  | 2 | 2 | 2 | 2 | 2 | 2 | 2 | 2 | No infection |
| 96 | Brown | Soft |  |  | 2 | 2 | 2 | 2 | 2 | 2 | 2 | 2 | No infection |
| 97 | Brown | Soft |  |  | 2 | 2 | 2 | 2 | 2 | 2 | 2 | 2 | No infection |
| 98 | Brown | Soft |  |  | 2 | 2 | 2 | 2 | 2 | 2 | 2 | 2 | No infection |
| 99 | Brown | Formed | *Crypto.* | *Crypto* | 2 | 2 | 2 | 2 | 2 | 2 | 2 | 1 | Single |
| 100 | Brown | Soft |  |  | 2 | 2 | 2 | 2 | 2 | 2 | 2 | 2 | No infection |
| 101 | Brown | Soft |  |  | 2 | 2 | 2 | 2 | 2 | 2 | 2 | 2 | No infection |
| 102 | Brown | Soft |  |  | 2 | 2 | 2 | 2 | 2 | 2 | 2 | 2 | No infection |
| 103 | Brown | Loose |  |  | 1 | 1 | 1 | 2 | 2 | 2 | 2 | 2 | Triple |
| 104 | Brown | Soft |  |  | 2 | 2 | 2 | 2 | 2 | 2 | 2 | 2 | No infection |
| 105 | Brown | Soft |  |  | 1 | 2 | 2 | 2 | 2 | 2 | 2 | 2 | Single |
| 106 | Brown | Soft |  |  | 2 | 2 | 2 | 2 | 2 | 2 | 2 | 2 | No infection |
| 107 | D. brown | Soft |  |  | 2 | 2 | 2 | 2 | 2 | 2 | 2 | 2 | No infection |
| 108 | Brown | Loose |  |  | 2 | 2 | 2 | 2 | 2 | 2 | 2 | 2 | No infection |
| 109 | Brown | Loose | *G. lamblia* |  | 2 | 2 | 2 | 2 | 2 | 1 | 2 | 2 | Single |
| 110 | Brown | Formed |  |  | 2 | 2 | 2 | 2 | 2 | 2 | 2 | 2 | No infection |
| 111 | Brown | Soft |  |  | 1 | 2 | 1 | 2 | 2 | 2 | 2 | 2 | Double |
| 112 | Brown | Soft |  |  | 2 | 2 | 2 | 2 | 2 | 2 | 2 | 2 | No infection |
